# Knowledge gap in vertebrate genetic diversity in a northern remanent of neotropical rainforest

**DOI:** 10.1371/journal.pone.0357894

**Published:** 2026-11-03

**Authors:** David José Martínez Cano, Víctor Hugo Reynoso, María de Lourdes Vázquez-Cruz, Edyciel Jordán Alvarado-Robledo, Yesenia Ithaí Ángeles-López, Eugenia Zarza

**Affiliations:** 1 Departamento de Ciencias de la Sustentabilidad, Unidad Tapachula, El Colegio de la Frontera Sur, Tapachula, Chiapas, México; 2 Departamento de Zoología/Pabellón Nacional de la Biodiversidad, Instituto de Biología, Universidad Nacional Autónoma de México, Ciudad de México, México; 3 Sceretaría de Ciencias, Humanidades, Tecnología e Innovación-Dirección de Innovación y Transferencia de Conocimiento, Benemérita Universidad Autónoma de Puebla, Puebla, México; 4 Secretaría de Ciencia, Humanidades, Tecnología e Innovación, Ciudad de México, México; National Cheng Kung University, TAIWAN

## Abstract

Los Tuxtlas Biosphere Reserve is one of the last remaining areas of primary tropical rainforest in Mexico. This fragile ecosystem heavily reduced by human perturbations is part of the Mesoamerican hotspot. The reserve harbors many endemic species. Thus, identifying, cataloging, and studying the biodiversity of this ecosystem has been of utmost importance. A promising approach for fast species identification to assess biodiversity is the use of DNA barcoding techniques. However, these techniques are limited by reliable DNA databases. Using novel Perl scripts, we generated a barcoding database to assess mitochondrial sequence availability in public databases for 903 vertebrate species that inhabit the reserve. Of these, 39 species had no sequences, 189 had complete mitochondrial genomes sequenced and the rest were partially sequenced. We studied over 80,000 sequences spanning 16 mitochondrial genomic markers and found 5 well-studied markers (COI-5P, CYTB, ND2, 16S rRNA and 12S rRNA) with more than 50% species coverage. The COI-5P region and CYTB were the best studied and most recommended markers for barcoding studies, and 7 additional markers have been preferred for one or more specific taxonomic groups. The remaining markers were almost exclusively sequenced as part of whole mitochondrial genomes. The best-known taxonomic group was mammals while the least known was reptiles. We also evaluated the sequencing frequency distribution for endemic, threatened and trade-restricted species driving interests in ecological research. In general, most species within each group were less sequenced when compared to the mean of all species, skewed by a few highly sequenced species of ecological interest. The most sequenced species are trade-restricted species, especially threatened species. This is driven by ecology-based research in the area. Finally, our results highlight a lack of barcoding research in local and endemic species of the Mesoamerican hotspot focusing on specific species but ignoring the wider range of biodiversity.

## 1. Introduction

Over the last decades, we have witnessed biodiversity loss at an alarming rate derived from human-driven changes in land use and overexploitation [1–4]. As a result, ecosystems have been destroyed to various degrees [4,5]. Estimates suggest that current biodiversity loss is 1000-fold times faster when compared to a natural background rate. This biodiversity loss crisis may result in the 6^th^ great mass extinction event in Earth’s history [2].Thinning off biodiversity can be catastrophic to life, thus a healthy balance between conservation and human use of resources is required [1,3,6,7]. To achieve correct management of natural resources and to support continued conservation efforts, it is vital to assess biodiversity and constant monitoring [3,4,8–10]. This is especially relevant in biodiversity hotspots, home to a high number of species and where the environment is under constant risk, threatening many rare or endemic species [1,3]. Mexico is a multi-diverse country with many biodiversity hotspots [1,11] for which conservation sites have been established. One such site is Los Tuxtlas Biosphere Reserve (LTBR) [12].

### 1.1. The Los Tuxtlas Biosphere Reserve

Mesoamerica is one of the original 25 global biodiversity hotspots. It ranges from the south of Mexico to Panama conforming a land bridge between North and South America and a land barrier between the Pacific and Atlantic Oceans. These geographical features facilitated the exchange of species between both continents allowing new endemic species to evolve [1,4]. Tropical rainforests are one of the most biodiverse ecosystems worldwide [4]. The LTBR is located in the southern mountain range of Veracruz, southeastern Mexico. It is the northernmost limit of the tropical rainforest in the American Continent and part of the Mesoamerican hotspot [13]. This site is considered a priority conservation zone, because it is one of the five zones with the greatest concentration of endemic species in Mexico, housing over 3,000 species of plants and over 1,500 species of animals [12,14–16]; however, this number can be underestimated considering newly revised inventories [17]. It covers 155,122 ha between 0 and 1,683 m above sea level from the Gulf of Mexico coast to the tallest volcano peak (San Martin Tuxtla), and the annual precipitation is 4,900 mm with seasonal variation [13,18]. The LTBR includes various ecosystem types, from tropical forests to mangroves and from cattle ranching pastures and agricultural terrains to human settlements. This creates a complex biological and sociocultural ecosystem [11,12,16,19], in which pollution and other anthropogenic activities have caused biodiversity loss [17,20–22].

Conservation efforts in this particularly fragile ecosystem dates from over half a century but deforestation still plagues this land, resulting in habitat loss and fragmentation that caused landscape degradation, and in turn, has negatively affected animal communities [11–13,18,23–25]. On the other hand, conservation efforts have seen a small (although sometimes temporary) increase in the tropical rainforest which suggests that not everything is lost [11,16,19,23].

### 1.2. Why barcoding?

At LTBR, species identification to record biodiversity has been mostly done using traditional methods based solely on morphological characters [10,13,24–26]. Identifying species this way can be complex because of several reasons, including the need for an expert taxonomist per biological group [27,28]. This expert needs to be capable of solving misleading factors such as cryptic taxa, traits with high phenotypic plasticity, various life stages and phenotypical differences between sexes, among others [27]; and cataloging errors may still occur [10,28]. Most used techniques are intrusive, perturbing the ecosystem with damage or death of the studied individuals [24]. While non-intrusive means of recording and cataloging biodiversity exist and are being used (e.g., video o sound recordings), they pose technical difficulties that may limit the number of organisms cataloged, primarily those “hard-to find” or cryptic species [25,28]. Nowadays, one novel, rapid, and reliable way to analyze the biodiversity in tropical ecosystems is through molecular approaches (i.e., DNA barcoding, metabarcoding of environmental DNA and bulk samples, and other omics disciplines) [26,29–31].

DNA barcoding focuses on delineating a genetic boundary and seeks comprehensive coverage to separate species [32]. DNA barcodes are small DNA sequences used to assign an organism to any taxonomic level (normally the species level) [33] by comparing it against a library of reference barcodes from previously identified and sequenced individuals [26,32].

DNA barcoding addresses many of the limitations related to morphological identification and enhances biodiversity inventories: it adds rigor to the identification process, can be automated and tested. It is faster, cheaper, more precise, scalable and can encompass larger datasets and thus species and interactions [27,34,35]. DNA barcoding coupled with high throughput sequencing, known as metabarcoding, has an important role in biodiversity assessment, as taxa can be detected from traces of DNA preserved in environmental (e.g., soil, water, air) or residual samples (e.g., hair, skin, skat) or other biological bioproducts [26]. This can be particularly useful for detecting elusive or endangered species, differentiating between cryptic species, or identifying species from unrecognizable samples like gut contents [27]. Barcoding and metabarcoding are useful in community ecology, functional trait evolution, trophic interactions, biodiversity conservation studies, policing illegally traded and endangered species, pest management, biosecurity, and healthcare [26,33,34]. Most importantly, DNA sequences are not prone to subjectivity and can be reanalyzed as technique improves or knowledge become available [27).

A reference barcode is not just a DNA sequence, it is the bridge between traditional and molecular means of identification. The sequence must be carefully chosen considering the taxonomical range and biology of the studied species, the sampling and end result that wants to be accomplished, and the existence of comparative data [26,31]. Then it requires proper sampling and vouchering of organisms (i.e., correct species identification and metadata recording). The generated sequences should be archived together with metadata in well-kept databases [32,35].

### 1.3. Building a barcoding reference database, challenges and current approaches

The generation of a barcoding reference database is one of the major challenges to attune DNA barcoding and taxonomy [26,31,36]. Two databases are commonly used as sources of reference sequences for barcoding: the GenBank and/or the BOLD database. GenBank (https://www.ncbi.nlm.nih.gov/), holds the largest DNA sequence database. However, because of its all-encompassing nature, records may not always have all the desired information due to different sequencing intents. The Barcode of Life Data Systems (BOLD: https://www.boldsystems.org/), was specifically created for DNA barcodes, usually from several individuals per species with complete taxonomic and voucher information. Thus, metadata is generally more complete, but sequences can be short and lack genomic context. It mostly contains data from the 658 base pairs mitochondrial region encoding the cytochrome c oxidase subunit 1 (known as COI-5P marker), considered the standard for metazoan organisms [27,28,32,35].

Unfortunately, databases are a work in progress, with many errors and omissions that reduce the quality of the information. These include: sequencing errors, sample contamination, mislabeling or misidentification, taxonomic issues (missing, wrong or outdated information), under and over representation, and lack of voucher specimens [27,36,37]. Databases can be improved by constant curation and by adding multiple entries to increase robustness, defined as the ability to generate a reliable identification even when faced with variations, to include natural allele variance. However, larger datasets and manual curation also have problems (e.g., higher computing power and storage needs and bioinformatics trained experts) [27,37].

Barcoding technology also has its own challenges. Fresh samples are not always available. Many techniques have been developed to use degraded DNA but they may yield relatively short sequences [27]. This issue can be offset using mini barcodes which target shorter DNA fragments but limit the study scope [31]. However, when generating a database from a repository, the data will likely include small, fragmented DNA resulting in multiple fragments within the same barcode and may even require some genomic context to be properly used [38]. Additionally, for some groups of organisms, specific barcodes may work poorly [39]. For example, COI-5P does not work well on plants and may work poorly on some invertebrates, amphibians, and reptiles [40]. Other gene regions have been used as barcodes to counter this, causing a lack of a unified barcode for the entire tree of life. As a consequence, various barcodes need to be tested for different taxa, and even within taxa, to fill knowledge gaps [33]. Barcodes spanning multiple markers like super or ultra-barcodes have been considered to solve this issue, but implementation is still challenging [36].

Mexico participated in the international barcode of life (iBOL, https://ibol.org/) BARCODE 500K program where many organisms were sequenced and barcodes generated [34,41]. Many species found in LTBR are of conservation, research, economic, and general interest and have found their way into strong sequencing efforts both nationally and internationally [42].

In this work, we aim to evaluate the barcoding knowledge gap and possible biases of multiple markers for all vertebrates found at the LTBR by retrieving mitochondrial gene sequences with potential use as barcodes. To do so, we developed a set of Perl scripts designed to build, manage, and analyze mtDNA sequence datasets. Additionally, we tried to demonstrate the capabilities of these scripts for automated data retrieval. We also aim to detect possible areas of improvement when implementing metabarcoding approaches by considering previous sequencing efforts and their driving interests in ecological research. Finally, stimulate to we would like to encourage to stimulate the scientific community to keep contributing to propagate the use of molecular techniques like DNA barcoding for species identification and monitoring of biodiversity, which seems to be under used.

## 2. Materials and methods

### 2.1. Checklist of species

We compiled a list of species expected to be present at LTBR drafted from the literature and curated using multiple approaches including literature review, consulting experts and searching on-line databases, to generate a comprehensive list of species and their synonyms properly spelled. This list was the base to retrieve the greatest number of related DNA sequences publicly available in both NCBI and BOLD databases. These databases do not always use the most recent taxonomic names, and sequences may be recorded as different species, but recent phylogenetic and taxonomic studies include them as synonymous. As such, we used the Enciclovida database (https://enciclovida.mx/), which is based on the CONABIO’s SNIB database (The National Commission for the Knowledge and Use of Biodiversity’s National System of Information for Biodiversity of Mexico database: https://www.snib.mx/) to add all synonym species names, curating the species list further and obtaining the relevant metadata, which included taxonomic information, common names, country of origin, presence status (exotic, native or endemic) and conservation status in international listings (CITES, or IUCN red list). To retrieve the information required by automated means we generated new Perl scripts in house (Fig 1).

**Fig 1:**
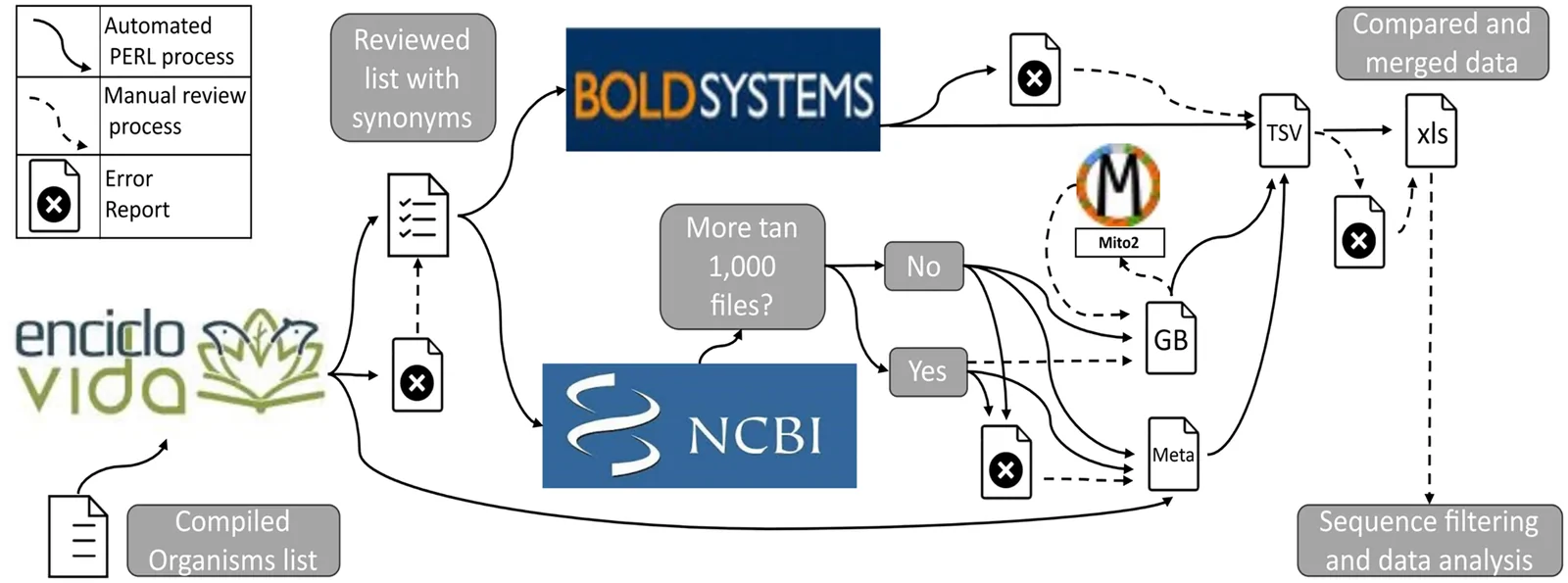
Workflow of data collection. The type of data obtained from each process is shown inside the file symbol; Meta = metadata, GB = GenBank, TSV = Tab separated values and xls = Excel.

### 2.2. Mitochondrial DNA search and data analysis

Historically, mitochondrial DNA (mtDNA) sequences were first used to reconstruct phylogenies and later for barcoding. Moreover, because of their small size, full mitochondrial genomes (mitogenomes for short) are constantly sequenced and there is a wealth of mtDNA sequences available that we use in barcoding studies. As such, we focused our efforts on searching and identifying mtDNA sequences. For both NCBI and BOLD, we developed Perl scripts, which also helped in obtaining, comparing, merging, and analyzing the desired information. To automatically retrieve information from NCBI as GenBank files we used E-utilitis, their in-house retrieval protocol tool and incorporated it into our scripts. Some species showed from a thousand to more than hundreds of thousands GenBank records. For these species we only retrieved between one to six of the whole mitogenome sequences to use as reference, considering subspecies and sampling location variety. When fewer than four whole mitogenomes existed, we recovered all of them.

Except for whole mitogenomes, most GenBank records downloaded had sequences that code for one or two feature tags per file (e.g., protein-coding genes, RNA genes, etc.). A normal vertebrate mitochondrial genome, i.e., a genome with a single copy per gene which also retains a stable mitochondrial architecture, includes 38 main feature tags: 13 protein coding genes, 2 rRNAs, 22 tRNAs and 1 control region; this is 37 genes plus a control region. The control region is not considered a gene and may be fragmented and placed in various loci within the genome. Other feature tags may be found in genomes like the origin of replication, but they are generally smaller, and they are not always reported in the file. Thus, sequences with multiple feature tags were first split into markers and each marker was grouped by its corresponding feature tag. For our analysis, we decided to ignore all tRNA sequences since most are short sequences, leaving us with 16 possible markers including 13 protein coding genes: cytochrome c oxidase subunits 1, 2 and 3 (COX1, COX2 and COX3), NADH dehydrogenase subunits 1, 2, 3, 4L, 4, 5, and 6 (ND1, ND2, ND3, ND4L, ND4, ND5 and ND6), ATP synthase 6 and 8 (ATP6 and ATP8), and cytochrome b (CYTB); 2 rRNA (12S rRNA and 16S rRNA) and a control region.

When necessary, sequences were annotated or reannotated (to increase specificity) using MITOS 2 web server (http://mitos2.bioinf.uni-leipzig.de/index.py), with their original annotation as a deciding factor if there was any choice to be made regarding the annotation. Finally, the corresponding metadata was assigned to each record.

BOLD records were obtained as TSV files which included both metadata and sequences. Each BOLD record revolves around a single marker and, in contrast to GenBank files, did not require further processing nor did their sequences require splitting. All data was then compared and merged and separated into various excel spreadsheets that can be used to generate the database (Fig 1). We also removed short sequences (<100 nucleotides, nt) and those belonging to tRNAs and other miscellaneous genetic features. All the data, including sequences, statistics, and data summaries are found in the supplementary data file (See S1 Table). All scripts can be obtained from github at: https://github.com/DJMC72/barcode-library-maker.

### 2.3. Statistical analyses

To analyze the distributions of the sequencing efforts from the species perspective (i.e., total number of records per species) we first made a boxplot for all species. To investigate possible biases due to conservation interests, we identified all LTBR vertebrate species listed as “nearly threatened” to “critically endangered” according to the Red List of Threatened Species of the International Union for Conservation of Nature (IUCN, https://www.iucnredlist.org/), those listed in the Convention on International Trade of Endangered Species (CITES, https://cites.org/eng), and those listed as endemic to Mexico according to the National System of Information for Biodiversity database (SNIB, https://www.snib.mx/). Using the resulting data, we produced boxplots for each species list to elucidate different distribution patterns between endemic, threatened (IUCN red list) and trade-restricted species (CITES). We also produce a boxplot for species that are both threatened and trade-restricted since these are generally expected to receive more attention and are likely sequenced more often. All the listings were retrieved indirectly from Enciclovida webpage (https://enciclovida.mx/) in September 2022.

To investigate if there is a difference in marker usage among taxonomic groups, we used the metadata obtained from the different databases to group the sequences by species and the species by taxonomic groups. Then we generated a matrix separating the sequences by marker (see S1 Table). We then calculated the number of species that have been sequenced per marker (species with at least one sequence of the corresponding marker) and summarized it. Further, we obtained the percentage of species with sequences for each group, thus weighting the data (number of species with sequences/total number of species) for each marker and taxonomic group combination. To differentiate active from passive sequencing (i.e., markers that are targeted for sequencing vs byproduct of whole genome sequencing) we generated a baseline for comparison which includes the species with sequenced mitogenomes plus those species for which the mitochondria has been mostly sequenced. This is to say, a portion of the mitogenome may be missing leading to a partial sequence but include at least 14 out of the 16 markers (we will refer to all species with 14–16 markers as “mostly sequenced species” or “MS species” for short) this sequencing baseline accounts for 28% of all species (See section 3.2).

The data robustness was estimated for each marker and taxonomic group combination as the total number of sequences (for that marker and taxonomic group) divided by the number of species with sequences. As above, to differentiate active sequencing and set a threshold for comparison, we used the average number of mitogenomes for each taxonomic group (total number of mitogenomes of the taxonomic group divided by the number of MS species of the same taxonomic group) (See section 3.2 and S1 Table) to estimate a robustness baseline.

When generating a reference database, we considered sequence completeness. As such, for each of the 5 most used markers and each species combination, we selected the longest sequence. For each marker, we calculated their average length for the taxonomic group by averaging the marker lengths reported as part of mitogenomes only (assuming these are the full-length reference sequences with an acceptable variance). We do this since not all species have fully sequenced mitogenomes. Finally, we normalized the data by calculating the completeness percentage of each species longest sequence relative to the average size in its taxonomic group. The resulting percentages were tabulated in increments of 10% and graphed (See section 3.2).

To identify the country where the sample originated, we separated the sequences by their locality metadata. Then, countries were further grouped by continents creating a matrix of species by country (see S1 Table and S3 Fig. The image was created with InteractiVenn [43]). We analyzed the resulting matrix and reported it. To make a more meaningful comparison, we recorded the species distribution by country for all 43 species of amphibians from amphibiaweb.org and iNaturalist.org (See S1 Table) and normalized the data. To normalize the data, we divided the number of sequenced species by the expected number of species according to its distribution for each country. We calculated the data robustness by country as the ratio of the total number of sequences by the number of species for each country (this is for all species) (See section 3.3).

## 3. Results

### 3.1. Data recovery

We compiled a list of a total of 903 vertebrate species for LTBR from which 120 were fishes, 43 amphibians, 118 reptiles, 485 birds, and 137 mammals (See S1 Table). From NCBI, we retrieved over 60,000 GenBank files from all 903 species, including 582 mitogenome sequences (Fig 2A). We recorded only 14 well-studied species showing more than 1,000 GenBank records, from which we retrieved up to six mitogenome sequences per species as reference. They include: two sea turtles, three food fishes, one ornamental fish, one migratory bird, and seven mammals including four bats (2 pests, 1 pollinator and 1 common in zoo displays), one game deer, one pet/research mouse and the coyote. All well-studied species have in common that they inhabit a wide range of countries and are of human interest. Finally, with MITOS2, we re-annotated 766 sequences (1.25% of all downloaded files).

**Fig 2:**
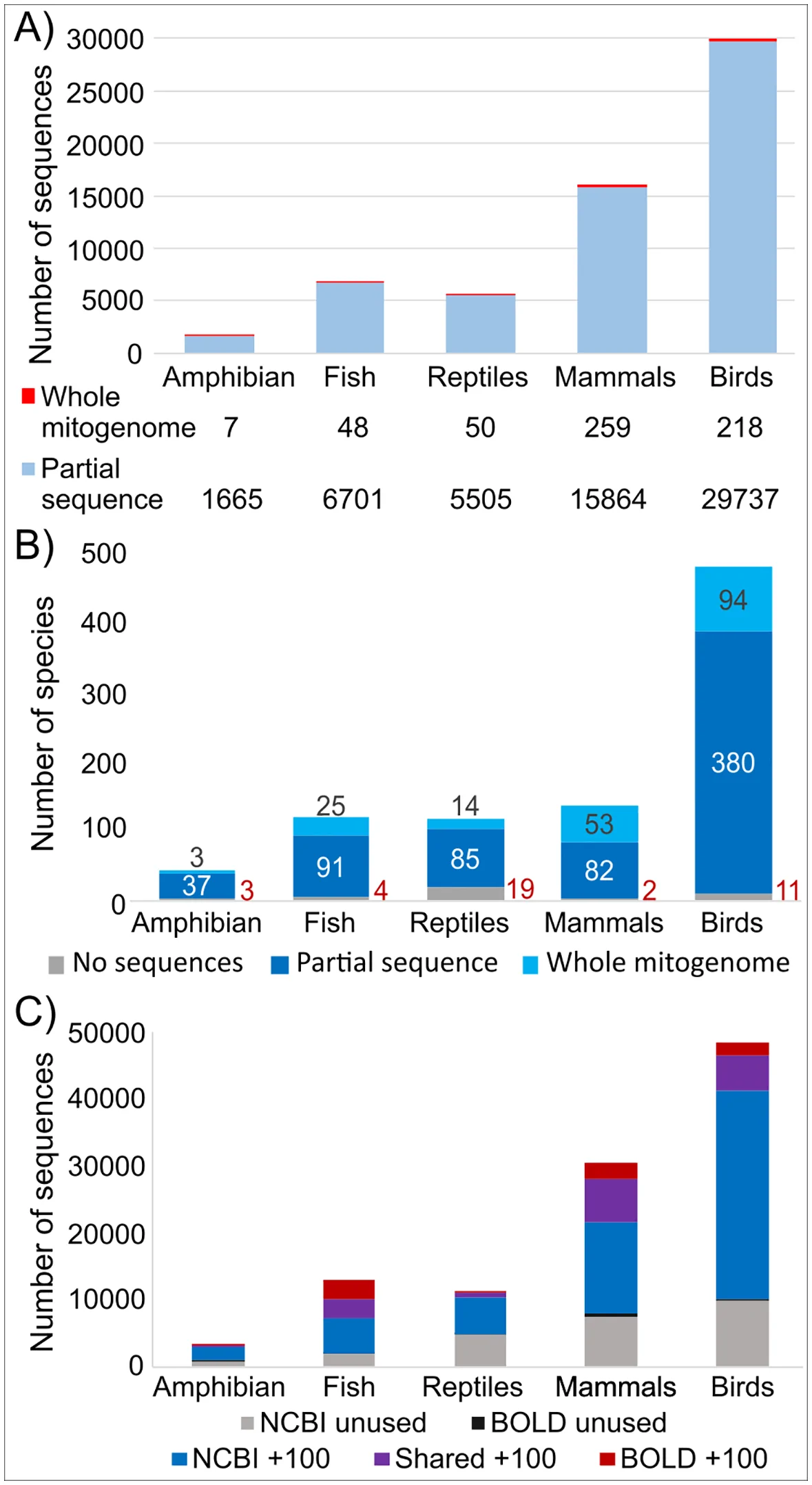
Statistics of downloaded sequences. A) Distribution of GenBank files in relation to the taxonomic classification. Red: proportion of mitogenomes. B) Number of species per taxa with complete, partial and no sequence. The number of species are as indicated: mitogenomes (black), partially sequenced genomes (white) and not sequenced (red). C) Distribution of marker sequences obtained by taxonomic group. Gray: sequences ≤ 100 nt long or that do not belong to the 16 markers studied (see section 2.2 for the full list); Black: records without nucleotide sequence; Red: sequences only found in BOLD; Blue: sequences only found in NCBI; Purple: sequences shared by both databases.

The 582 mitogenome sequences corresponded to 189 species or 20.9% of all species studied (Fig 2B). Mammals showed the largest proportion of mitogenomes (38.7%) while amphibians showed the smallest proportion (6.9%). Also, we were unable to find GenBank records for 39 species (4.3% of all species studied), of which reptiles had the most species with missing records (16.1%) and mammals the least (1.4%). All other species showed at least one partial sequence of the mitochondrial genome.

From the downloaded GenBank files we extracted 98,262 single-marker sequences for all markers. From BOLD we downloaded 23,674 records, of which 362 records lacked the nucleotide sequence and were removed from the analysis leaving 23,312 single-marker sequences. Since BOLD also includes some sequences mined from NCBI we compared and combined all sequences and obtained 106,146 single-marker sequences including 15,428 sequences shared by both databases. After removing the sequences with fewer than 100 nt and other small sequences like tRNAs we obtained 80,806 sequences (Fig 2C).

### 3.2. Sequencing knowledge gaps

We grouped the obtained sequences by marker and found that COX1 is the most frequently sequenced marker, comprising nearly 29% of all sequences, followed by the control region (18%), CYTB (17%), ND2 (11%), 16S (4%), and 12S (3%). All other 10 markers shared the remaining 17%. When separating the data by taxonomic group, we found different distributions (Fig 3).

**Fig 3:**
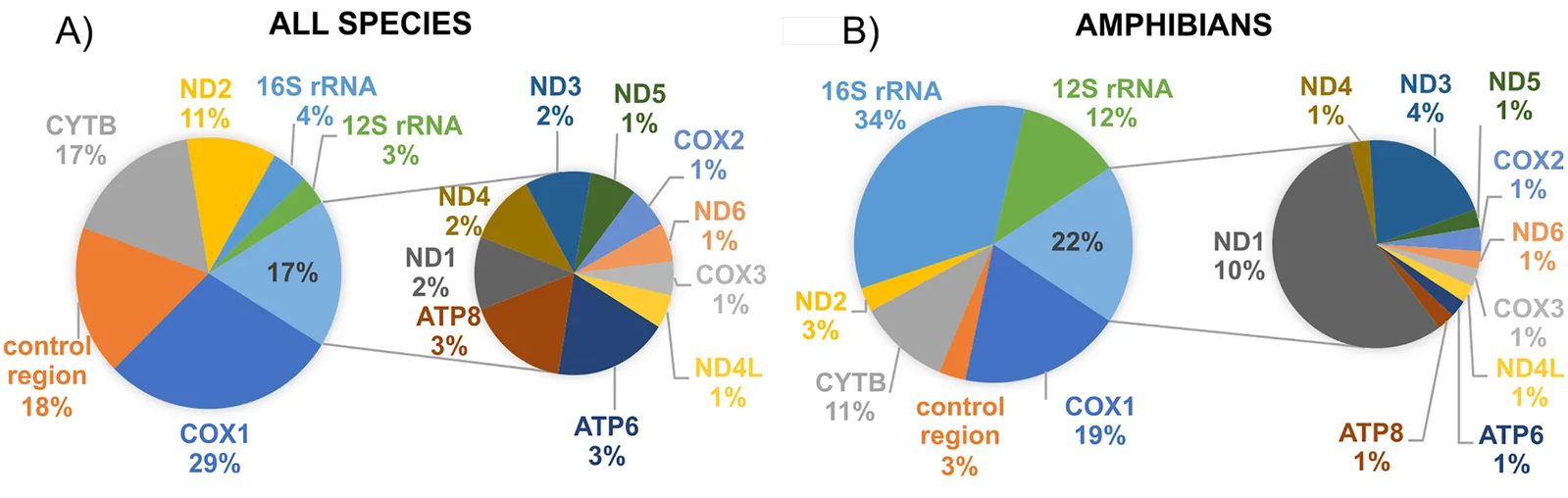
Distribution of sequences by marker. A) for all species; B) for amphibians (for other taxonomic groups please see S2 Fig).

We calculated the number of species per marker that have been sequenced (Fig 4). Only 5 of the 16 markers are sequenced in more than half of the studied species (Fig 4a). Similarly, these markers are COX1 (779 species or 86.3%), CYTB (759 species or 84.1%), ND2 (627 species or 69.4%), 12S (527 species or 58.4%) and 16S (493 species or 54.6%). The remaining markers ranged from 381 to 261 species (42.2 to 28.9%).

**Fig 4:**
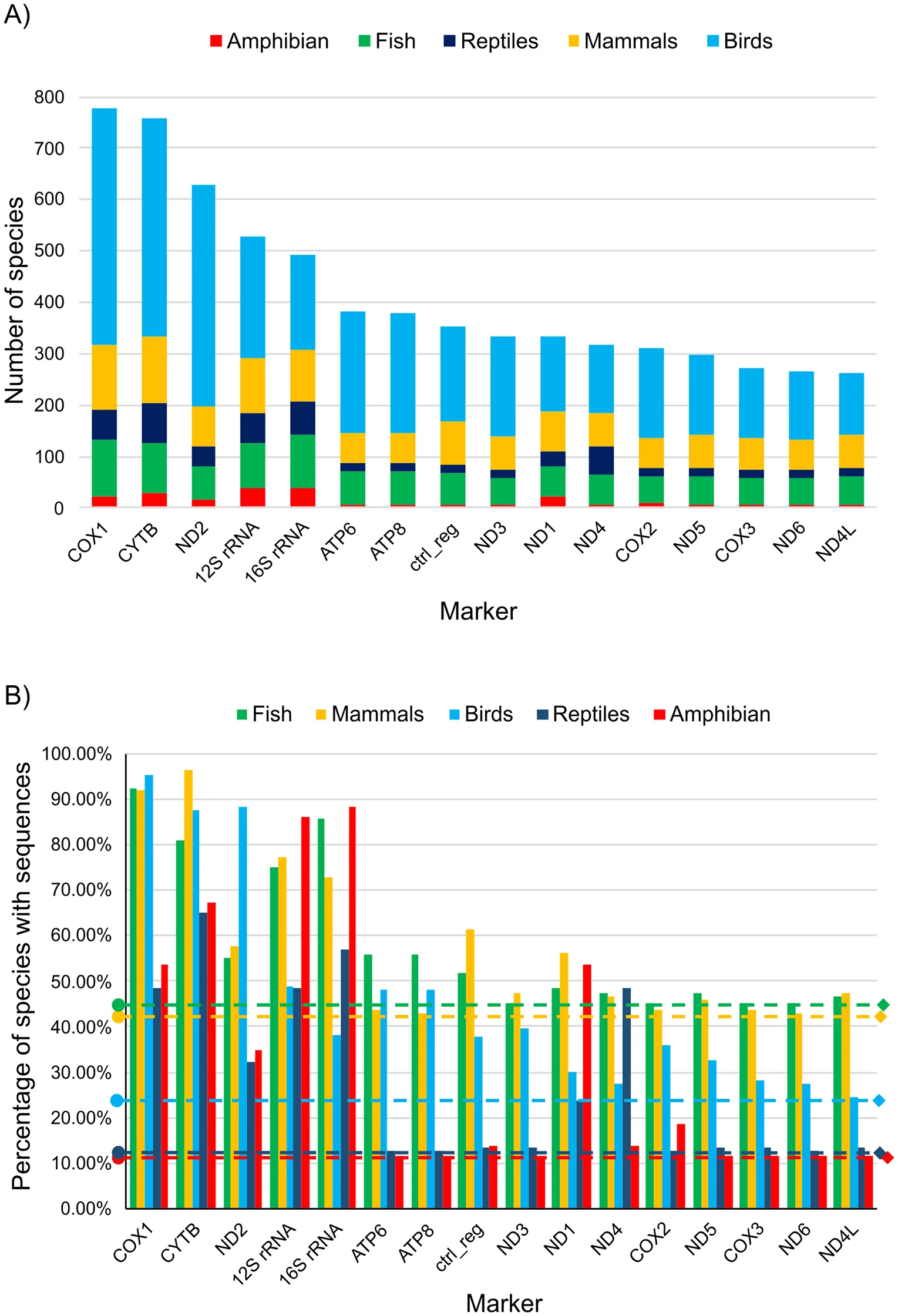
Number of species with one or more sequences per marker. A) overall count of species separated by color for each taxonomic group, B) percentage of species with sequences for each marker separated by taxonomic group for comparison. Dotted lines represent a passive sequencing baseline for each taxon.

To differentiate passive from active efforts in sequencing, we used mostly sequenced (MS) species as a baseline (i.e., species with 14 out of 16 markers sequenced). Thus, anything beyond the baseline is an active effort to sequence a specific marker. In total, the baseline accounts for 28% of all species, and by taxonomic group they comprise 11.6% of amphibians, 45.0% of fishes, 12.7% of reptiles, 42.3% of mammals, and 24.1% of birds (Fig 4b). As such, ND4 is a well-studied marker in reptiles, while ND1 is for amphibians. ND1 also shows a larger percentage of sequenced species than the baseline in mammals and reptiles. COX2 and ND3 show a larger percentage for birds. ATP6 and ATP8 show a larger percentage for birds and to a lesser degree for fish. The control region shows a larger percentage for mammals and birds. ND5, COX3, ND6 and ND4L have received nearly no interest as markers since they are at or close to the baseline, and they have been sequenced almost exclusively as part of mitogenome sequencing projects. Sequencing efforts are clearly outlined for our top 5 markers (COX1, CYTB, ND2, 12S and 16S). For each of these markers we see a much larger percentage in all taxonomic groups and in some cases the percentage of species with sequences reaches over 80%.

Data robustness for ND5, COX3, ND6 and ND4L is very similar to the robustness baseline values, indicating, that these sequences are derived from mitogenomes. Such robustness baseline represents: ~ 2 homologous mitogenomes per species for fish, amphibians, and birds, 3.5 for reptiles and 5 for mammals.

The control region, COX1 and CYTB are widely used in all groups, and the average number of homologous sequences are at least 3.4 times larger than the robustness baseline for all groups. Similarly, although for specific taxa only, ND4 has 3.86 times the average number of homologs than the robustness baseline in reptiles. ATP6 and ATP8 have about 3 times the average number of homologous sequences than the robustness baseline for birds and about 2.5 times for fish. ND1 has nearly 4 times the average number of homologous sequences in amphibians and 5 times in reptiles. ND3 shows 2.2 times the average number of homologous sequences for birds. Finally, ND2 shows a slightly higher average number of homologous sequences for most groups but over 7 times the robustness baseline value for birds and 5 times for reptiles. (Fig 5).

**Fig 5:**
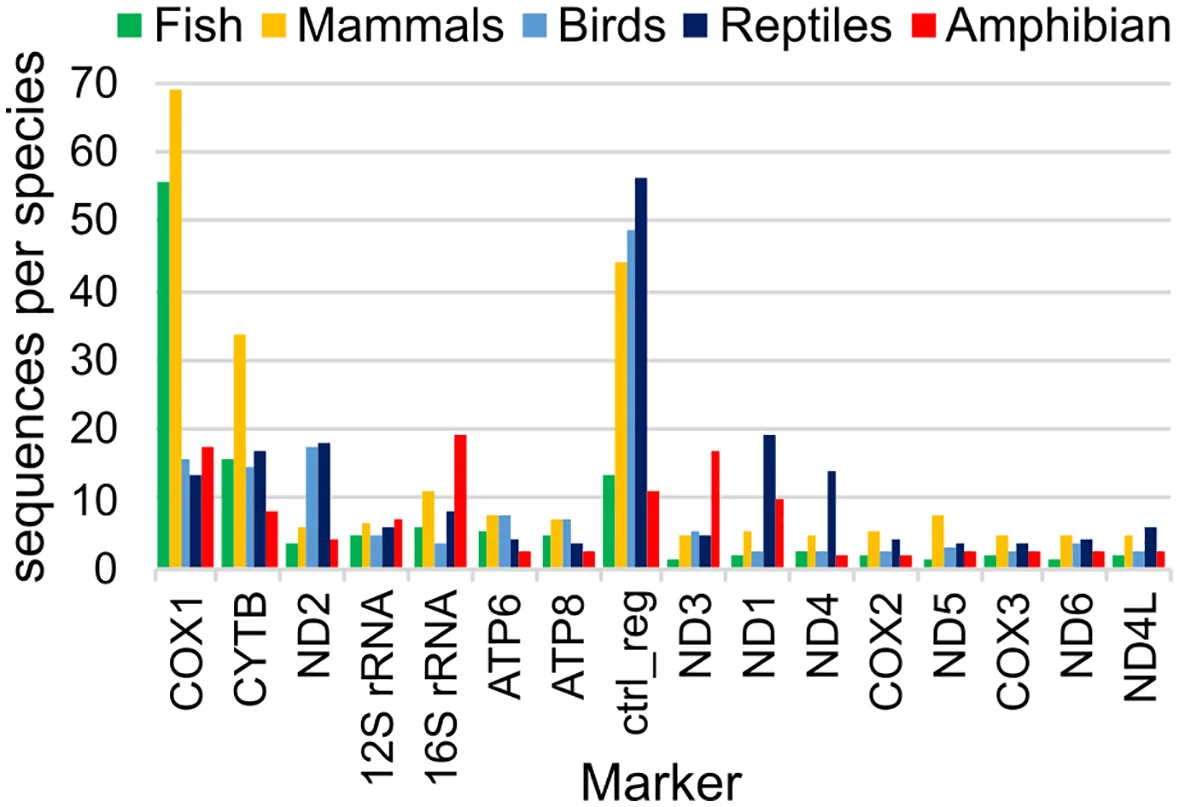
Average number of homologous sequences per sequenced species for each of the 16 markers.

The knowledge gap is also evident in sequence completeness (Fig 6). ND2 has the most contrasting transitions from dark to white showing mostly 2 shades. Only 21 species had partial reference sequences for ND2. Thus, knowledge gap mostly comes from non-sequenced species. COX1 shows a contrasting transition between 3 shades with 467 species showing partial reference sequences mostly in the 40–50% size bracket (380 species). CYTB, 16S and 12S have between 144–169 species having partial references sequences but are less contrasting with a smoother, more gradual transition this is especially visible in CYTB for amphibians, 12S for reptiles and fish and 16S for reptiles, amphibians and fish. Thus, we are missing sequence information since there is a considerable number of partial sequences in our reference database.

**Fig 6:**
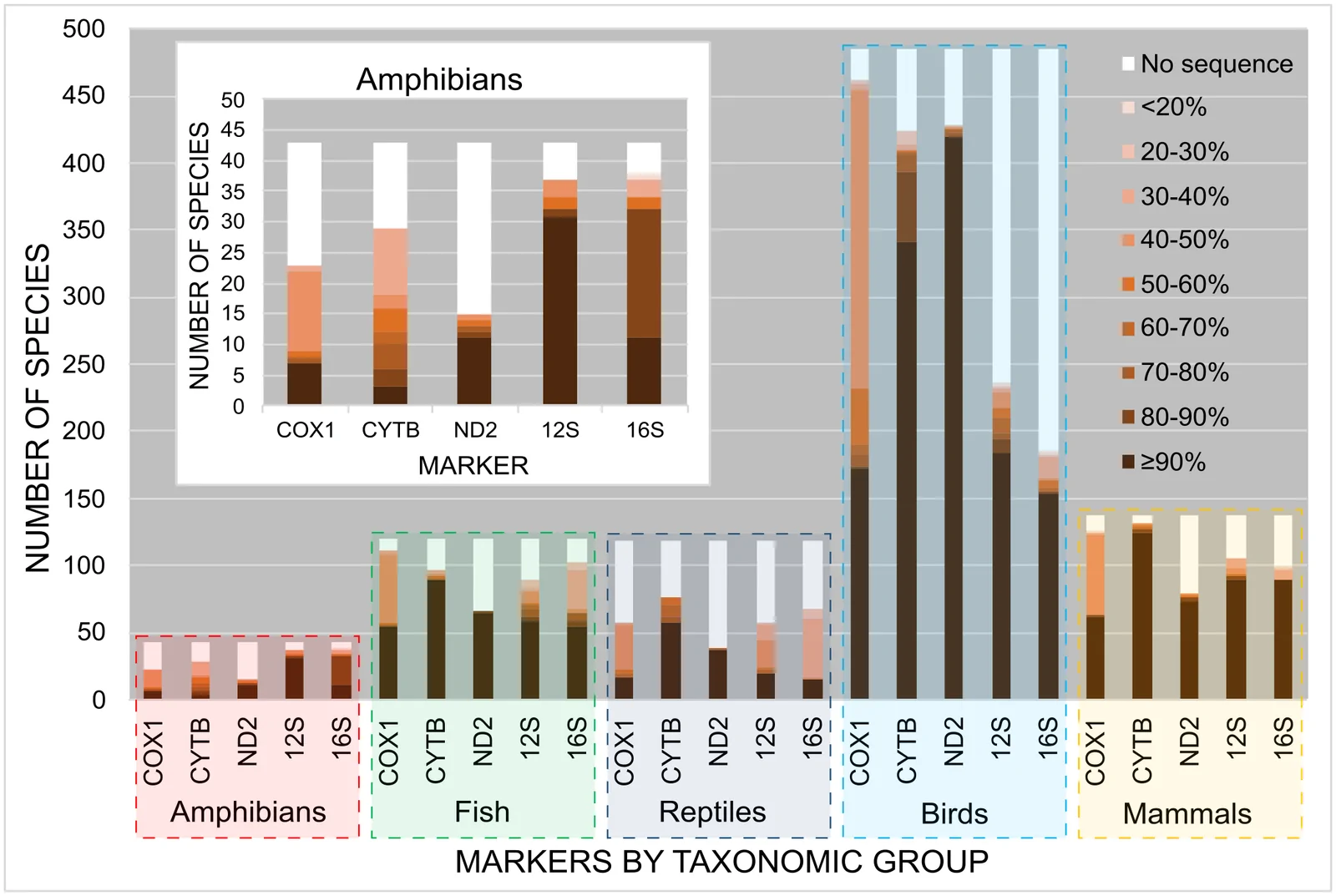
Distribution of the number of species by reference sequence completeness when compared to taxa average by marker. The fraction in white is the number of species that have not been sequenced. Lighter colors represent species with partial reference sequence. In the top left corner is a closeup of amphibians for clarity.

### 3.3. Sequencing effort in species of ecological interest and regional interest

High interest ecological focus groups can be studied to understand driving efforts in sequencing in the local community. The mean for all 903 species was 89.49 sequences per species, and the median was only 36 sequences (Fig 7). Thus, the data is strongly skewed most sequences concentrated in a few species as homologous sequences.

**Fig 7:**
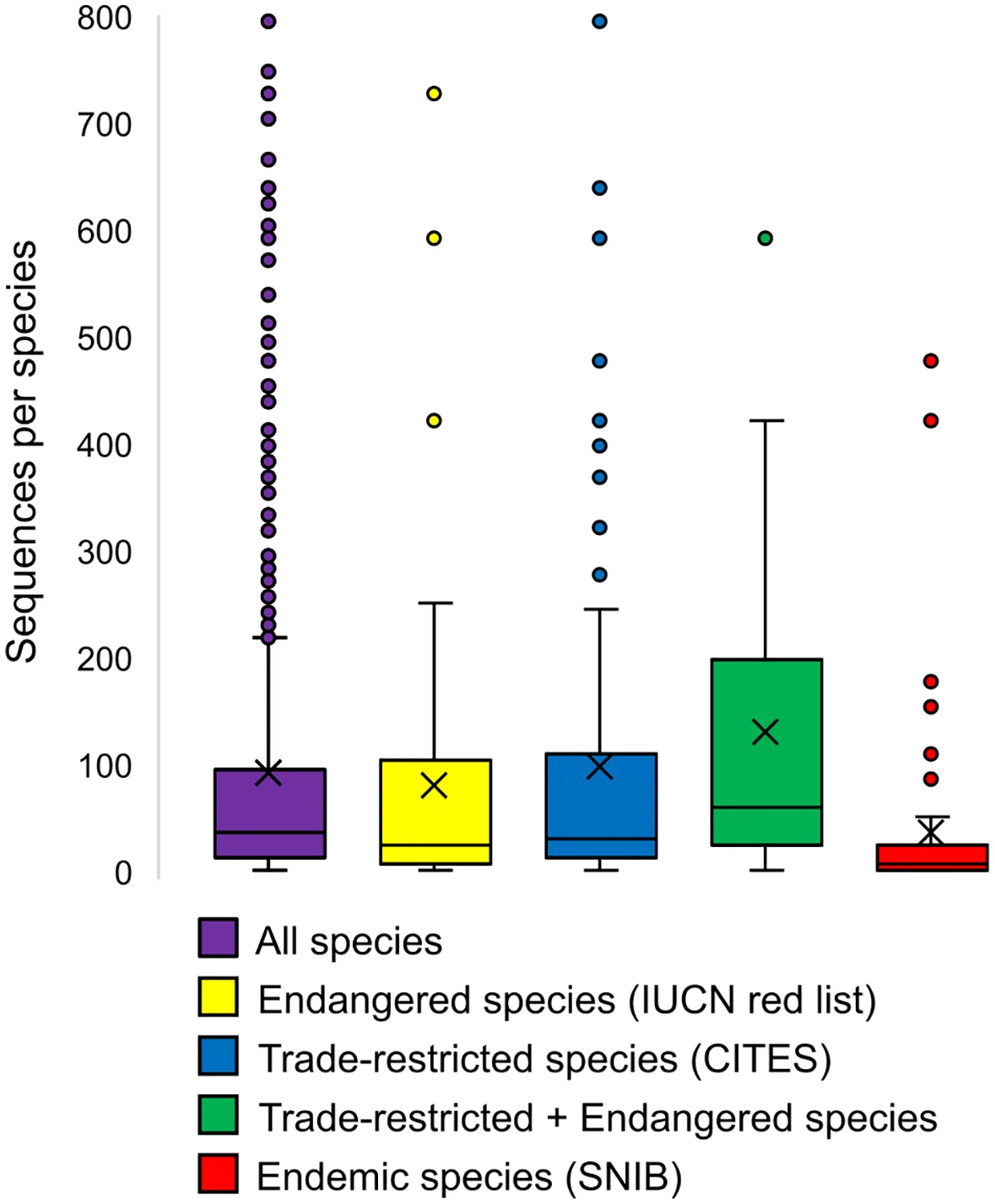
Boxplots of the total number of sequences per species. Endangered species are species listed in the IUCN red list as “nearly threatened” to “critically endangered”; trade-restricted species are species listed in any of the CITES appendixes, and endemic species are species recognized as endemic by the local authorities (SNIB).

A similar mean was observed for the 71 species listed in the IUCN red list as “nearly threatened” to “critically endangered” (from now on referred to as “endangered species”) and the 117 species listed in CITES appendices (from now on referred to as “trade-restricted species”). Endangered species mean decreased by 10.5% to 80.1 sequences per species when compared to that of all species, and trade-restricted species mean increased by 7% to 95.8 sequences per species (Fig 7). Meanwhile, the median was reduced in both cases to 23 sequences (36.1%) and 28 sequences (22.2%) respectively. Twenty-two trade-restricted species are also endangered. The mean and median values for these species were 129.3 and 59.5 sequences per species respectively (Fig 7), with all of them having at least one sequence. Thus, different interests generate an additive effect resulting in higher probabilities of being sequenced.

The study of endemic species reflects the use and management of local natural resources. In our list, 59 species were endemic to Mexico according to the SNIB database. Most endemic species have very few sequences, with a mean of only 35.69 sequences per species and a median of 6 sequences, showing a data distribution considerably different from previous ecological focus groups (Fig 7). We found that 15 endemic species are not sequenced at all, this means that 38.5% of all LTBR species with no sequences in Genbank or BOLD are endemic species as opposed to 4 trade-restricted species (10.25%, 1 of which is endemic) and 6 endangered species (15.38%, 5 of which are endemic). This suggests that the effect is likely not due to random sampling.

From these results, we suspected that the sequenced populations may not be local. Out of the over 80,000 obtained sequences, only 46,661 (57.74%) had locality information (see S1 Table). Based on that information, 813 species (90% of all species) were sequenced from organisms within the American continent. Of these, 96 species were also sequenced from organisms from other continents. Europe was the most represented continent and Oceania the least (see S3 Fig). Most of these species are birds, marine fishes, and sea turtles and many have migration patterns mainly towards Europe and Africa. Moreover, some species have been introduced to other continents (both from and to the American continent) as pets (e.g., common green iguana – *Iguana iguana*), display animals in zoos or circus (e.g., Jaguar – *Panthera onca*), for consumption or as bait (e.g., Nile tilapia – *Oreochromis niloticus*) and other reasons like attempts of biocontrol (e.g., marine toad – *Rhinella marina*) (for example [44],).

The largest number of sequenced LTBR species were sampled from Mexico (409 sp.), USA (376 sp.), Panama (301 sp.), Brazil (204 sp.) and Canada (191 sp.). Other countries had less than 140 species sequenced. The contribution of Mexico to our list of LTBR sequenced species is only 45.29% (at least one sequence per species of any marker), suggesting indeed a lack of sequences from native populations. If sequences from all countries of the Mesoamerican Biodiversity Hotspot [4] are included, the number of sequenced species only increases to 68.32% (617 species). Interestingly, if we add together the three countries with the most sequenced species (Mexico, USA and Panama) the number of sequenced species reaches 81.62% (737 species).

## 4. Discussion

### 4.1. The pipeline

Starting in 2012 [45], barcoding knowledge gap studies worldwide have increased substantially, especially in the last 5 years [46–55]. Nowadays, datasets can become quite large limiting most studies to a combination of specific taxa and locations, promoting the use of bioinformatic tools. Barcoding knowledge gap studies are normally focused in cryptic, less known, rare or hard to sample species like insects [46–49] or aquatic animals [50–54] in well-known location; or general species overviews and commonly barcoded species in little-known locations [55–57].

BLIM (Barcode Library Maker), our pipeline, was intended to be more exploratory than other tools, testing most of the mitochondrial genome for potential barcodes or combination of them while still capable of producing a barcoding library. Some tools used for this purpose already exist. For example, the R packages “worms” [58] uses the World Register of Marine Species database to obtain synonymous and current species names; “bold” [59] can retrieve BOLD data; and “rentrez” [60] can retrieve data from NCBI and has been used previously in knowledge gap studies [50,52,53]. All of these tools can only retrieve raw data, but data sorting and some other analyses were still mostly done manually. Other software like DataTaxa [61], a metadata retrieval software; or dataFishing [62], a tool for mining data from various databases, do sort the metadata but were not meant for barcoding studies. Others like BOLD.R [63] and BOLDconnectR [64] provide metadata but only related to BOLD. Many other tools create comprehensive barcode databases but are either mostly focused on single markers like MIDORI2, CO-ARBitrator, MARES, COInr, or require significant computational resources like CRUX, rCRUX MetaCurator, Metaxa2 and CRABS [65].

Although our study could be considered a preliminary test of BLIM, it was able to retrieve the data and sort out the metadata needed. The software is capable of downloading about ~40 sequences per minute with an internet speed of ~150 Mbps. This software is a diagnostic tool that encompasses identification of synonym names, sequence retrieval, and metadata sorting features. BLIM’s performance is data dependent, influenced by size and quality of input data and data availability. Thus, it is challenging to compare its performance to other existing software designed with intended different purposes (e.g., datataxa) or that do not explore multiple genes (e.g., COInr) or that heavily depend on computational resources (e.g., CRUX). BLIM aims at finding which data is missing and help in decision making without using too many resources. We believe that a shift in focus towards alleviating knowledge gaps in datasets needs to be encouraged and tools need to be developed. BLIM is still a work in progress, but we believe it will evolve into a useful tool to explore possible barcodes, assess knowledge gaps and make a preliminary barcoding library in a few quick steps from your laptop.

### 4.2. Sequencing knowledge gap in Los Tuxtlas

Previous knowledge gap studies on Mexican biodiversity were done using GBIF and literature data [66], but not directly from barcoding data as we have done here. This provides insight into the state of the data itself. We found many errors and missing data, to the point that over 1% of the sequences had to be re-annotated. We also found several errors when comparing BOLD and NCBI shared records. For example, NCBI accession: DQ434506 shows changes in coordinates in latitude and longitude.

There is a considerable knowledge gap for most markers. Of the 903 studied species, only 189 were completely sequenced, 39 were not sequenced at all, and the rest were partially sequenced. Five markers were the most used (COX1, CYTB, ND2, 16S, and 12S). Only a few species have the control region sequenced even though it is one of the most sequenced markers (see below). ND1, ND3, ND4, COX2, ATP6 and ATP8 were preferentially studied for one or more taxonomic groups and ND5, COX3, ND6 and ND4L were mostly unstudied, with sequences derived directly from mitogenomes.

We found similar patterns for data robustness. COX1 and CYTB are sequenced for over 84% of the species with many homologous sequences underling them as the best-established markers used in all taxa [67,68]. We found similar observations for ND4 in reptiles; ND1 in amphibians; ATP6 and ATP8 in birds and fish; and ND2 and ND3 in birds.

A few peculiarities were also observed. Reptiles show five times the average number of ND2 and ND1 homologous sequences than the robustness baseline although few species have been sequenced suggesting these markers are used mostly in a subset of species (lizards and the snake *Nerodia rhombifer,* respectively). In fact, if we remove the sequences from this snake as an outlier, the average number of homologous sequences is only 0.5 times more than the robustness baseline for ND1 in reptiles. Similarly, most of the homologous sequences of ND3 in amphibians are from the toad *Rhinella marina*. In short, robustness in these taxa only relate to a few species. Likewise, the control region accounts for 18% of all sequences but it has only been sequenced in 38.5% of all species, probably because it has been used as a specialized tool to identify specific reptiles (sea turtles and crocodiles), amphibians (*Rhinella marina*) and fish species (e.g., *Archosargus probatocephalus, Cynoscion nebulosus, Micropogonias undulatus, Syngnathus scovelli,* and *Xiphophorus hellerii*). Over 60% of mammal species are sequenced for this marker. Interestingly, most of the species without sequences are bats and rodents.

The opposite is also true, where marker use is more extensive and data robustness is relatively low. This is the case for COX2 in birds and for 16S and 12S that have been sequenced for a relatively large set of species. For 16S and 12S the number of homologous sequences per species is rather low for nearly all taxonomic groups (except for 16S in amphibians). This is likely associated with the use of these markers in phylogenetic studies where one sequence per species per study is a common practice [39,67]. Other uses, such as species delimitation, identification, or quality control [33], are more likely to repeat the sequencing of different individuals from the same species. In amphibians, 16S is a prominent DNA barcoding marker to complement COI-5P in species identification [42].

Partial reference sequences were also common in four of the five best studied markers. The lack of complete references can affect the performance of the database during taxonomic assignments. Ideally, when generating a database, we want most species to have a sequence encompassing the full length of the gene. These partial reference sequences were especially observed in markers that are less robust like 12S or 16S, or poorly studied groups like reptiles, amphibians and fish [66]. Marker use can also affect this. COX1 is extensively used as a barcode, but rarely fully sequenced (~1550 nt). Instead only short fragments are normally obtained with the COI-5P region (657 nt standard length) becoming the most frequently sequenced marker [40]. Thus, in the data we see a bimodal distribution, where one mode represents the species barcoded for the COI-5P region, and the other represents the species sequenced for the whole COX1 gene (mostly from whole mitogenomes). This could also be considered a knowledge gap of sorts since we lack a reference sequence for COX1 in more than half the species. Currently this might not be a problem as high-throughput sequencing technologies are biased towards shorter sequences [31,69]. However, as long-read sequencing technologies develop and become more accessible, it will not be possible to take advantage of the full potential of complete COX1 sequences in future studies.

### 4.3. Interest driving sequencing

The knowledge gap is influenced by marker preference which dictates sequencing focus to cater for different purposes and interests for different taxa. This may be due to biological, technical or even personal preferences. For example, COX1 contains the standard barcode for metazoans (COI-5P) [27]. 16S and 12S are widely used in broad-range phylogenetic studies because of their lower mutation rate and high sequence conservation [67,70] while CYTB is commonly used in species delimitation because it is less conserved [67,71]. ND2 has a higher mutation rate, and it is sometimes used to complement CYTB in species delimitation. In birds, ND2 has also been used in wide-range phylogenetic studies [71–73].

Personal preferences drive marker choice because information accumulation results in perpetuation of this preference [68]. For example, in our study, 12S and 16S appear to be under-sequenced when compared to the other markers, both in regards to the total number of sequences (4 and 3% of all the sequences respectively, Fig 3a) and species sequenced (Fig 4a) which seems to be related to sequencing biases driven by birds.16S has been sequenced for 88.37% of amphibian species (most sequenced marker), 85.83% of fish species (2^nd^ most), 56.78% of reptile species (2^nd^ most) and 72.99% of mammal species (3^rd^ most) (Fig 4b). However, for birds, that account for more than half of the total number of species included in our study, 16S is the 7^th^ most sequenced marker with only 38.14% of the species sequenced. 12S shows similar results while the opposite occurs with ND2. Thus, we speculate that ND2 was preferred as a marker over 16S and 12S in some studies in birds.

We were particularly keen in assessing the interest driving ecological research like conservation, economic exploitation, and endemism. However, because our data set is based on a biosphere reserve for which most concerns are ecological, we did not study in depth other common drivers of research but they can be represented in some of the best-studied species like those of economic (e.g., display animals and pets), agricultural (e.g., fishes and pollinator bats) or medical value (e.g., coyote and pest bats) [74].

When reviewing the sequencing frequency distribution for three groups of ecological interest, we found that only a few species are highly sequenced underlying the uneven treatment to different species [75]. The means of endangered and trade-restricted species remained close to, or even higher than the mean for all species, even while showing a larger reduction of the median indicating that the groups are less sequenced in general, denoting the importance given to these few highly sequenced species within these groups. Among these, we find some commercially important and endangered species like the great cats, marine turtles or the many species that can be traded as pets like iguanas, and some species of parrots, falcons and frogs. On the other hand, endemic species mean dropped bellow the median for all species, and one quarter of the species had no sequences, suggesting less interest in these species by the local scientific community which may reflect a much broader general trend.

### 4.4. Integrating Genetic Barcoding into Local Biodiversity Assessments: Potential and challenges

Roughly 57% of the sequences studied here had locality data and accounted for 813 species. All of which had at least one sample originated from the American continent and only 45% of the species had samples from Mexican populations, suggesting indeed a lack of sequences from native populations.

To provide a more visual example of what this result entails, we obtained the country distribution for amphibian species in our study (amphibiaweb.org and iNaturalist.org, see S1 Table) and cross referenced with the locality data for comparison (Fig 8). Mexico is the 9th overall country and the 3rd Mesoamerican hotspot country with the highest percentage of LTBR amphibian species sequenced. Although, this is mostly due to two well-studied widely distributed species (the toad *Rhinella marina* and the frog *Trachycephalus typhonius*) that make up to half of the species per country observations in non-Mesoamerican countries. Nonetheless, in Mesoamerican countries, the percentage of sequenced LTBR amphibian species ranged from 61% to 12.5%, clearly indicating an underrepresentation of native populations and biodiversity.

**Fig 8:**
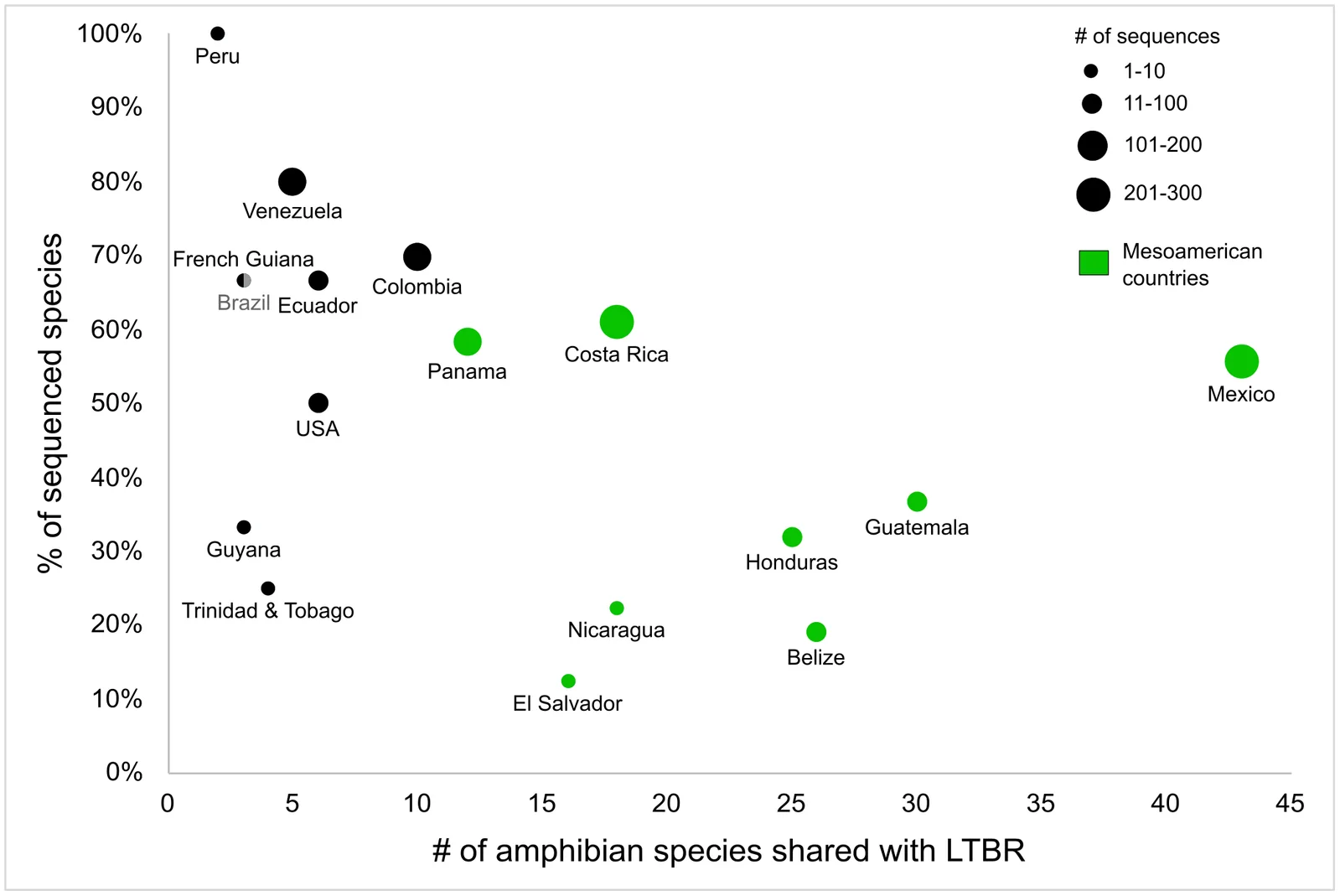
Two-dimensional plot of shared LTBR amphibian species vs percentage of sequenced species per country. Only American countries and territories with at least one sequenced species were included. Overlapping values are colored in grey (i.e., Brazil). The percentage of sequenced species for Mexico includes four endemic species which did not have locality data.

We also reviewed the sequences per species per country for amphibians (Fig 9). México showed 19.26 sequences/species, the second-best value for all countries, while other Mesoamerican hotspot countries showed considerably low values, between 9 to 4.5 sequences per species. This suggests that despite some shortcomings there is an increasing interest in the molecular approach to study local ecosystems [76].

**Fig 9:**
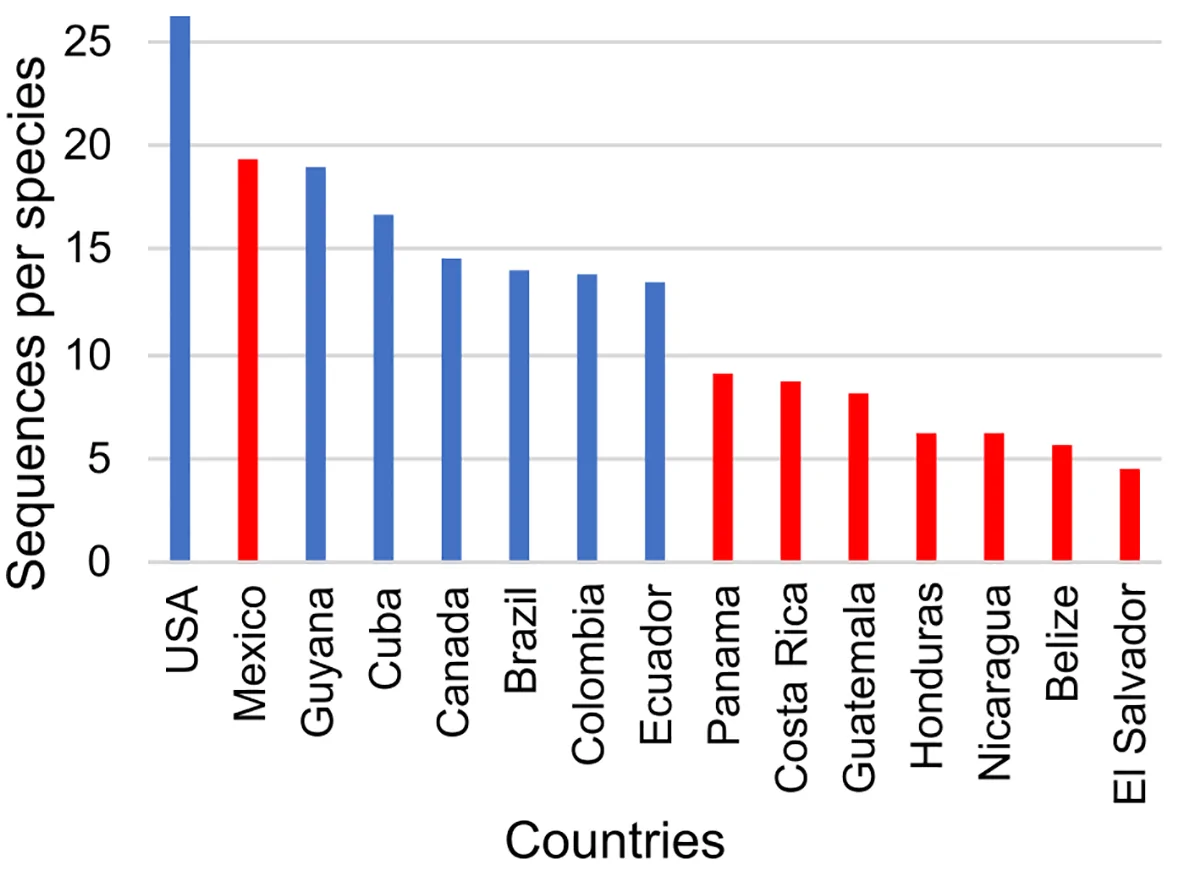
Number of sequences per species for each country. We report the top 8 countries and territories followed by the countries which are part of the Mesoamerican hotspot (in red).

Interest drives sequencing and these results give us a glimpse on the lack of interest towards the use of sequencing tools for the study of native species biodiversity. We cannot rule out other aspects that may impact sequencing, like the precarious conservation state of some populations which make finding specimens increasingly difficult, lack of funding, relatively high sequencing costs, lack of facilities and technology, and political and governmental roadblocks [77,78]. However, when comparing recent research spending, Mexico is the 4^th^ country in the American continent showing most spending with 4.8 billion USD allocated to research in 2023 after USA (932 billion USD in 2022, also the most spent by a single country worldwide), Canada (37 billion USD in 2023) and Brazil (17 billion USD in 2020) [79,80]. Although Mexico spends a considerable amount of funding in research every year [76], the budget is still less than one third of the budget spent in Brazil, and at the same time almost ten times more than the budget for six of the seven other Mesoamerican countries combined (490 million USD in various years from 2015 to 2022, no data obtained for Belize) [79,80].

It is important to make a clear distinction between the astounding sequencing effort by some sectors of the scientific community to study the species in Mexico and the huge potential yet to be unearthed of sequencing as a tool for taxonomic assignment required in biodiversity studies, for which comprehensive wildlife research is also paramount. Several whole genome sequencing projects have been completed in Mexican institutions, mostly related to conservation, medical and agriculturally important species, but genome sequencing of wildlife species do not seem to be a priority [74]. Additionally, when compared, our result agrees with other knowledge gap studies which showed that tropical countries were understudied relative to temperate countries, and similarly lower income countries had less studies overall with most of the studies being focused on vertebrates and specially mammals and birds with reptiles, amphibian and freshwater fish being less studied [57,66,81]. We need to remember that a barcode is not just a sequence. It is a tool for taxonomic assignment linked to invaluable physical evidence and metadata normally of multiple individuals within the population to account for natural variation. When we use repurposed sequences, we would likely lack information that was not collected or deposited in the database which may impact the quality of the study. Similarly, with new discoveries, taxonomic assignment is a constantly changing field. Without a source (i.e., a physical voucher) and accompanying metadata the repurposed sequence could eventually be set aside because it is no longer able to identify a species. Unfortunately, most of our sequences are repurposed and generally few per marker and per species. This can be best observed from the number of sequences found in the BOLD database intended as barcodes (in red) compared to the repurposed sequences from NCBI (purple) which far outnumber them (fig 2). In short, while overall sequencing efforts are advancing by leaps and bounds, biodiversity studies have barely scratched the surface of what is currently possible with molecular tools, although it is moving in the right direction there is still much to be done.

### 4.5. Implications for metabarcoding studies and biomonitoring

Despite the knowledge gaps in the four most studied markers, we believe there is enough data and sequences to use them in barcoding endeavors especially for both CYTB and COI-5P. Both had more than 80% species coverage and some robustness that can improve our identification while including population variation. 16S and 12S RNAs could also be used, but the reduced coverage of birds may be a concern and thus are less recommended. Other markers can be very useful for specific taxonomic groups like ND2 for birds or ND4 for reptiles. We also recommend when using barcodes to keep in mind various aspects like species coverage, sequence completeness and locality. All these qualities improve accuracy during taxonomic assignment. Although we do not minimize the value of sequences coming from elsewhere, we believe that sequences from specimens in the same area of interest are more likely to allow proper identification at the species level, and may prevent much hardship in delimiting species identity [82]. We believe that a great effort is needed in sequencing all species locally. On site reference genetic databases are necessary to precise the taxonomic status of a lineage in the face of the constant change of species limits distribution [37].

## 5. Conclusions

In summary, of the 16 markers, five markers are well-studied and one more is frequently sequenced but biased towards a subset of organisms. The other 10 are mostly unstudied with 6 markers being studied for only specific taxonomic groups. Thus, there is a considerable knowledge gap for most markers. Selecting the right marker for a study depends not only on the amount of available sequence, but also on their original purpose and inherent limitations. Therefore, we must thoroughly understand existing knowledge gaps before launching any metabarcoding study.

Our study gives us a glimpse into the current state of the art but also into the research interest and policies which have shaped sequencing for the last few decades. Agricultiural, economical, and conservation interest are the main driving forces for ecology-based research which correlates with the current concerns related to fragmentation in the LTBR. Finally, our results highlight a lack of barcoding research in the Mesoamerican hotspot.

Bioinformatic tools are needed to reduce workload, improve data quality and optimize cumbersome tasks. In this work, we also developed the pipeline BLIM for accessing barcoding data, summarize metadata and find errors between GenBank and BOLD records. Our database can become a valuable resource for future sequencing and metabarcoding endeavors. It can help find knowledge gaps to be filled, to improve native species sequencing, and generate robust data. This is particularly important within natural protected areas where non-invasive monitoring (e.g., based on metabarcoding and environmental DNA) is needed.

## Supporting information

S1 Table Supplementary data. Raw data and statistical data used in this article.
(XLSX)

S2 Fig Distribution of sequences by marker. A) for mammals; B) for birds; C) for fish; D) for reptiles.
(DOCX)

S3 Fig Venn diagram showing LTBR species sampled outside of the American Continent. The numbers denote the number of species while its placement denotes the set of continents where this species has been found, sampled and sequenced. Image created with InteractiVenn.
(TIFF)
