# Supplementary material for: Knowledge gap in vertebrate genetic diversity in a northern remanent of neotropical rainforest
Source: PLoS One. 2026 Nov 3;21(11):e0357894. doi: 10.1371/journal.pone.0357894 (PMC13730339; doi:10.1371/journal.pone.0357894)
Supplement: Supplementary file 1 [file pone.0357894.s002.docx]

A)

B)

C)

D)
